# Characteristics of Hospitalized Acute Q Fever Patients during a Large Epidemic, The Netherlands

**DOI:** 10.1371/journal.pone.0091764

**Published:** 2014-03-10

**Authors:** Cornelia C. H. Wielders, Annemarie M. H. Wuister, Veerle L. de Visser, Monique G. de Jager-Leclercq, Cornelis A. R. Groot, Frederika Dijkstra, Arianne B. van Gageldonk-Lafeber, Jeroen P. G. van Leuken, Peter C. Wever, Wim van der Hoek, Peter M. Schneeberger

**Affiliations:** 1 Department of Medical Microbiology and Infection Control, Jeroen Bosch Hospital, ’s-Hertogenbosch, the Netherlands; 2 Centre for Infectious Disease Control, National Institute for Public Health and the Environment (RIVM), Bilthoven, the Netherlands; 3 Department of Internal Medicine, Bernhoven Hospital, Uden, the Netherlands; 4 Department of Pulmonary Diseases, Jeroen Bosch Hospital, ’s-Hertogenbosch, the Netherlands; 5 Department of Pulmonary Diseases, Bernhoven Hospital, Uden, the Netherlands; 6 Institute for Risk Assessment Sciences, Faculty of Veterinary Sciences, Utrecht University, Utrecht, the Netherlands; University of Minnesota, United States of America

## Abstract

**Background:**

From 2007 to 2009, the Netherlands experienced a major Q fever epidemic, with higher hospitalization rates than the 2–5% reported in the literature for acute Q fever pneumonia and hepatitis. We describe epidemiological and clinical features of hospitalized acute Q fever patients and compared patients presenting with Q fever pneumonia with patients admitted for other forms of community-acquired pneumonia (CAP). We also examined whether proximity to infected ruminant farms was a risk factor for hospitalization.

**Methods:**

A retrospective cohort study was conducted for all patients diagnosed and hospitalized with acute Q fever between 2007 and 2009 in one general hospital situated in the high incidence area in the south of the Netherlands. Pneumonia severity scores (PSI and CURB-65) of acute Q fever pneumonia patients (defined as infiltrate on a chest x-ray) were compared with data from CAP patients. Hepatitis was defined as a >twofold the reference value for alanine aminotransferase and for bilirubin.

**Results:**

Among the 183 hospitalized acute Q fever patients, 86.0% had pneumonia. Elevated liver enzymes (alanine aminotransferase) were found in 32.3% of patients, although hepatitis was not observed in any of them. The most frequent clinical signs upon presentation were fever, cough and dyspnoea. The median duration of admission was five days. Acute Q fever pneumonia patients were younger, had less co-morbidity, and lower PSI and CURB-65 scores than other CAP patients. Anecdotal information from attending physicians suggests that some patients were admitted because of severe subjective dyspnoea, which was not included in the scoring systems. Proximity to an infected ruminant farm was not associated with hospitalization.

**Conclusion:**

Hospitalized Dutch acute Q fever patients mostly presented with fever and pneumonia. Patients with acute Q fever pneumonia were hospitalized despite low PSI and CURB-65 scores, presumably because subjective dyspnoea was not included in the scoring systems.

## Introduction

Q fever is a zoonosis caused by the intracellular bacterium *Coxiella burnetii*, which can be transmitted from animals (mainly goats and sheep) to humans [1]. The incubation period ranges from two to three weeks, and the clinical presentation of acute Q fever is diverse: from flu-like illness to pneumonia or hepatitis [1], [2], [3]. Geographical differences are reported in clinical presentation. Patients in the Netherlands [4], [5] and the Basque region of Spain [6] experience more pneumonia, while data from France [2], [7], [8] and southern Spain [9] show more cases of hepatitis. Clinical features of acute Q fever include: fever, headache, fatigue, cough, night sweating, dyspnoea, chest pain, nausea, vomiting, diarrhoea, and joint and muscle pain [2], [3], [5], [10]. A *C. burnetii* infection can also remain asymptomatic. Both symptomatic and asymptomatic *C. burnetii* infections can evolve into chronic Q fever [11]. Chronic Q fever is difficult to diagnose and involves serological testing as well as a thorough clinical evaluation [12].

From 2007 to 2009, the Netherlands experienced a major acute Q fever epidemic with over 3,500 notified cases, which mainly occurred in the southern part of the country [13]. Q fever-infected dairy goat farms were established as the source of the epidemic in the Netherlands and living close to an infected farm was identified as a strong risk factor for acquiring acute Q fever [14]. The percentage of notified acute Q fever patients being hospitalized was 46.1% in 2007, decreasing to 21.1% in 2008 and 19.5% in 2009, with an overall average admission rate in these three years of 21.6% [4]. Previous studies from France reported a hospitalization rate of only 2–5% for acute Q fever pneumonia and hepatitis [2], [3]. There are no explanations for the high admission rate in the Netherlands, though it has been suggested that a high dose of *C. burnetii* bacteria or prolonged continuous exposure to the bacterium might lead to a more severe clinical presentation [15], [16], [17].

The objectives of this study were: (1) to describe the clinical and epidemiological features of patients hospitalized for acute Q fever in the Netherlands in the period 2007–2009; (2) to compare the clinical presentation of hospital-admitted patients with acute Q fever pneumonia with patients with other forms of community-acquired pneumonia (CAP); (3) to compare the proximity to an infected ruminant farm as a measure of infection dose between hospitalized and non-hospitalized patients.

## Methods

### Ethics statement

The medical ethical committee of Bernhoven Hospital (‘Comissie Ethiek’) approved this study. Patient information was anonymized and de-identified prior to analysis. According to Dutch legislation, written consent from each individual patient was not required because of the retrospective nature of this study and the use of anonymized information.

### Study design and setting

A retrospective cohort study was conducted with patients hospitalized for acute Q fever from 2007 to 2009 in Bernhoven Hospital (locations in Oss and Veghel, Noord-Brabant, currently located in Uden), which is located at the centre of the Dutch Q fever epidemic area. Data were collected from the clinical patient files and the laboratory information system.

### Hospital admission

All patients living in the hospital's catchment area who were hospitalized with acute Q fever at Bernhoven Hospital from January 2007 to December 2009 were included in the study. Acute Q fever-related hospitalization was defined as hospital admission of one or more nights at Bernhoven Hospital, either within one month before or after microbiological diagnosis of acute Q fever, or within one month before or after the onset of symptoms retrospectively attributed to acute Q fever based on microbiological diagnostics, which were carried out after the patient was discharged. Relevant medical staff were consulted when it was uncertain whether the patient was hospitalized for the acute Q fever infection or for another concurrent medical condition. When patients were admitted for Q fever-related reasons on two or more occasions within the defined period, clinical information was collected for the first admission only.

Exclusion criteria were (1) non-Q fever-related hospitalizations of patients who had an acute Q fever infection; (2) presence of antibodies against *C. burnetii* found during hospitalization but no IgG phase II antibodies found during follow-up; and (3) presence of proven chronic Q fever at the time of hospitalization, defined as a positive polymerase chain reaction (PCR) result in combination with an IgG phase I titre≥1∶1,024 measured by immunofluorescence assay (IFA; Focus Diagnostics, Inc., Cypress, CA, USA).

### Microbiological diagnosis of acute Q fever and other infections

For the microbiological diagnosis of acute Q fever we used four categories, based on PCR results as described previously [18] and on serology (Table 1). Standard microbiological assays including culture, serology, PCR and urinary antigen tests were performed for detection of other respiratory pathogens (e.g., *Streptococcus pneumoniae, Mycoplasma pneumoniae, Legionella pneumophila*). All microbiological tests for Bernhoven Hospital were conducted by the Department of Medical Microbiology and Infection Control of the Jeroen Bosch Hospital in ’s-Hertogenbosch.

**Table 1:** Laboratory case definition of acute Q fever patients included in the study (n = 183).

No.	Laboratory definition	n (%)
1	PCR positive and an IgG phase II and/or IgG phase I seroconversion with at least IgG phase II≥1∶64 in follow-up	46 (25.1)
2	IgG phase II and/or phase I seroconversion with at least IgG phase II≥1∶64 in follow-up	59 (32.2)
3	IgG phase II and/or IgG phase I fourfold increase in antibody titre with at least IgG phase II≥1∶64 in follow-up	9 (4.9)
4	IgM phase II “positive” (untitrated)^a^ or ≥1∶64 and IgG phase II “positive” (untitrated) or ≥1∶64 at diagnosis, and IgG phase II≥1∶64 in follow-up (when available)^b^	69 (37.7)

No.: number.

a Due to the large number of samples that were submitted to our laboratory for Q fever diagnostics (over 18,000 samples in 2009), IFA was sometimes not titrated, but only recorded as “positive”.

b At least one follow-up sample was available for 177/183 (96.7%) patients.

### Clinical data and follow-up

Patient demographic characteristics, clinical symptoms, medical history, laboratory and radiological findings, additional microbiological tests, and treatment details were retrospectively collected from the clinical patient files and the laboratory information system (January–June 2013). Data on clinical features and laboratory findings was at first contact at the time of admission and also included information on medical history. Data on follow-up, such as mortality, development of chronic Q fever, and other long-term effects of the disease, were collected for two years after the date of admission.

Additionally, the CURB-65 score (Confusion, blood Urea nitrogen, Respiratory rate, Blood pressure, age≥65) [19] and the Pneumonia Severity Index (PSI) [20] were determined. Unavailable features of the scores (e.g., respiratory rate or pH) were counted as being normal. The CURB-65 score ranges from 0 to 5, while the PSI is divided into classes I to V. CURB-65 score≥2 and PSI≥IV were defined as severe pneumonia requiring hospitalization. Hepatitis due to acute Q fever was defined as a >twofold increase in the reference value for alanine aminotransferase (ALT; >90 U/L for males, >70 U/L for females) in combination with a >twofold increase in bilirubin (>34 µmol/L). Relative bradycardia was defined as body temperature >38.9°C and heart rate <120 beats/min without the use of beta-blocker medication, pacemaker-induced rhythms, or arrhythmias [21]. Adequate antibiotic treatment was defined as doxycycline (200 mg/day), moxifloxacin (400 mg/day) or ciprofloxacin (1,000 mg/day per oral dose) [22]. The definition of chronic Q fever was made according to the classification of the Dutch Q fever Consensus Group, which uses an IgG phase I titre≥1∶1,024 measured IFA as cut-off [12].

### Pneumonia patients

An analysis was performed to compare the clinical presentation and pneumonia severity of acute Q fever pneumonia patients (defined as an infiltrate observed on a chest x-ray) with non-Q fever-related pneumonia patients, by using data from a study performed from November 2007 to January 2010 in the neighbouring Jeroen Bosch Hospital, ’s-Hertogenbosch (which is 19 km from the Bernhoven Hospital) [23]. This control group consisted of patients attending the emergency department with CAP, defined as an acute symptomatic infection of the lower respiratory tract which developed outside the hospital or nursing home, whereby a new infiltrate is demonstrated on a chest x-ray [24]. Patients diagnosed with acute Q fever and patients that were not hospitalized after visiting the emergency department were excluded from this database. A separate analysis was performed for pneumonia patients with microbiologically proven bacterial aetiology.

### Environmental exposure

The second analysis was performed to study the role of the extent of environmental exposure, by measuring the distance in metres (m) between the nearest infected farm and the patients' home address (postal code). Both hospitalized and non-hospitalized acute Q fever patients diagnosed between 2007 and 2009 and living in the catchment area of Bernhoven Hospital were included. Distances were categorised according to Schimmer *et al.* [14]: 0–2,000 m, >2,000–5,000 m, and >5,000 m. Small ruminant farms that experienced abortion waves (defined as >5% abortions of all pregnant animals) caused by *C. burnetii* and bulk tank milk positive farms were considered infected (data provided by the Animal Health Service and the Food and Consumer Product Safety Authority).

### Statistical analyses

Descriptive characteristics, medical history, symptoms, radiologic findings, laboratory tests results, treatment, and follow-up were investigated by calculating relative frequencies, and median and interquartile ranges (IQRs). Prevalence of underlying disease and health status of admitted patients were compared with prevalence data of the general population in the same region (Municipal Health Service, Hart voor Brabant), and of the entire country based on data from Statistics Netherlands, the National Public Health Compass, and the National Cancer Registry [25], [26], [27].

For the analysis of pneumonia and other CAP patients, relative frequencies, and median and IQRs were calculated. Chi-square tests, Chi-square tests for trend, and Mann-Whitney U tests were used to test for differences between acute Q fever pneumonia and either CAP patients or other bacterial pneumonia patients. In these tests, a *p*-value <0.05 was considered statistically significant. Odds ratios (ORs) and 95% confidence intervals (95% CI) were also calculated. For the environmental exposure analysis, median distances for hospitalized and non-hospitalized patients from their home address to the closest infected small ruminant farm were calculated and the Mann-Whitney U test was used to test for statistical significance. Logistic regression was performed for the analysis of the environmental exposure with the distance category >5,000 m used as a reference. Data were analysed using IBM SPSS Statistics version 19.0.0 (SPSS Inc.).

## Results

### Characteristics of hospitalized patients

From 2007 to 2009, 1,728 Q fever patients living in Bernhoven Hospital's catchment area were diagnosed with Q fever. Within one month before or after diagnosis and/or onset of symptoms, 208 of these 1,728 (12.0%) acute Q fever patients were hospitalized. These rates in the hospital's catchment area differed largely during the epidemic years: 40/79 (50.6%) in 2007, 81/684 (11.8%) in 2008, and 87/965 (9.0%) in 2009. Twenty-five patients were excluded: 22 because of a non Q fever-related reason for hospitalization despite having acute Q fever, two because of undetectable IgG phase II antibodies during follow-up, and one because of hospitalization with a proven chronic Q fever infection, leading to a hospital admission rate of 10.6% (183/1,728). The 183 hospitalized acute Q fever patients comprised of 114 males (62.3%) with a median age of 54 years at admission (range 4–86; two patients <18 years) and a median length of admission of 5 days (range 1–53) (Table 2). Hospitalized patients were significantly older than the non-hospitalized patients living in Bernhoven Hospital's catchment area (55 vs. 48 years; *p*<0.001), and no significant difference was found for the gender distribution. All patients except one were admitted following a visit or referral by the general practitioner (GP) to the emergency department.

**Table 2:** Baseline characteristics of hospitalized acute Q fever patients from 2007 to 2009 (n = 183) compared with the general Dutch population in the region and nationwide.

	Hospitalized acute Q fever patients (n = 183)	Prevalence (%) in the study area (MHS Hart voor Brabant)^a^	Prevalence (%) in the Netherlands^a^
	n (%)	%	%
Male	114 (62.3)		
Median age at admission [IQR] (years)	54 [41–65]		
Current smoker	82 (47.1)^b^	26.4	26.3
History of smoking	38 (22.8)^c^		
Median duration of admission [IQR] (days)	5 3–7		
Median duration between onset of illness and admission [IQR] (days)^d^	4 3–7		
**Medical history**			
COPD/asthma	29 (15.8)	7.8	7.7
Heart failure	21 (11.5)		0.7^e^
Diabetes mellitus type I	2 (1.1)	0.8	0.9
Diabetes mellitus type II	17 (9.3)	3.4	3.4
Malignancy	14 (7.7)		3.2^f^
Heart valve insufficiency	12 (6.6)		
Aortic aneurysm	10 (5.5)		
Vascular prosthesis	9 (4.9)		
Autoimmune disease	9 (4.9)		
CVA	7 (3.8)	3.3	2.4
Heart valve prosthesis	5 (2.7)		
Chronic renal failure	5 (2.7)		
Liver disease	4 (2.2)		
No underlying condition	109 (59.0)		

COPD: chronic obstructive pulmonary disease; CVA: cerebrovascular accident; IQR: interquartile range; MHS: Municipal Health Service.

a Unless otherwise indicated, data extracted from Statistics Netherlands (CBS), 2008–2011: http://statline.cbs.nl/statweb/(website accessed 2013 July 30) [27].

b Information missing for nine cases.

c Information missing for 18 cases.

d Information missing for three cases.

e Prevalence in 2007, data extracted from National Public Health Compass: http://www.nationaalkompas.nl/gezondheid-en-ziekte/ziekten-en-aandoeningen/hartvaatstelsel/hartfalen/cijfers-hartfalen-prevalentie-incidentie-en-sterfte-uit-de-vtv-2010/(website accessed 2013 July 30) [26].

f 20-years prevalence in 2009, data extracted from Comprehensive Cancer Centre the Netherlands (IKNL): http://www.cijfersoverkanker.nl/(website accessed 2013 July 30) [25].

### Microbiological diagnosis of acute Q fever and other infections

Table 1 presents the number of patients that fulfilled each of the categories of our case definition. Additional microbiological tests and their results are presented in Table 3. A sputum sample was obtained in 42 (23.0%) of patients. Nine patients had no leucocytes (21.4%), 22 had few leucocytes (52.4%), a moderate number of leucocytes was found in eight (19.0%) patients, and only three (7.1%) patients had numerous leucocytes in their sputum sample. One patient had pneumonia infection caused by *Coxiella burnetii* and *Legionella pneumophila*. Detectable *Mycoplasma pneumoniae* antibodies (≥1∶40) were found in 22 patients (17.9% of patients in whom the specific test was performed), although only two patients met our local definition of a *M. pneumoniae* infection (antibody titre≥1∶320).

**Table 3:** Additional microbiological tests and outcomes in hospitalized acute Q fever patients.

Microbiological sample collected or test performed	Hospitalized acute Q fever patients (n = 183)	Outcomes	
		Negative/not abnormal	Positive
	n (%)	n (%)	n (%)
Sputum sample	42 (23.0)	40 (95.2)^a^	0 (0.0)
Blood culture	140 (76.5)	138 (98.6)	2 (1.4)^b^
*Mycoplasma pneumoniae* serology	123 (67.2)	101 (82.1)	2 (1.6)^c^
*Legionella pneumophila* urinary antigen test or serology	103 (56.3)	102 (99.0)	1 (1.0)
*Streptococcus pneumoniae* urinary antigen test	10 (5.5)	10 (100)	0 (0.0)

a No pathogens cultured; in two patients sputum had been sampled which was not suitable for cultivation.

b *Staphylococcus epidermidis* (both are probably a contamination of the sample).

c *Mycoplasma pneumoniae* antibodies ≥1∶320, 20 additional patients had detectable antibodies: 1∶40 (n = 8), 1∶80 (n = 4), 1∶160 (n = 8).

### Clinical data and follow-up

Fever was the most reported symptom (80.9%) among hospitalized acute Q fever patients, followed by cough (50.8%) and dyspnoea (47.5%) (Table 4). Pneumonia with unilateral or bilateral infiltration on a chest x-ray was observed in 154/179 (86.0%) patients with an x-ray available (Table 5). Moderate to severe pneumonia based on a CURB-65 score≥2 was seen in 34 (22.1%) acute Q fever pneumonia patients, while PSI≥IV was found in 27 (17.5%). A total of 16 (10.4%) patients had high PSI and CURB-65 scores, and combining both scores resulted in 45 (29.2%) patients with a moderate to severe pneumonia infection. Anecdotal information from attending physicians suggests that some patients were admitted because of severe subjective dyspnoea. Adequate antibiotic treatment was started during or after hospitalization in 84.7% of the patients (Table 5). Relative bradycardia was observed in 35/36 patients with a body temperature >38.9°C in the absence of the use of beta-blocker medication, pacemaker-induced rhythms, or arrhythmias.

**Table 4:** Symptoms at time of admission of hospitalized acute Q fever patients (n = 183).

	Hospitalized acute Q fever patients (n = 183)
Symptoms	n (%)
Fever (≥38.0°C)	148 (80.9)
Cough	93 (50.8)
Dyspnoea	87 (47.5)
Malaise	81 (44.3)
Fatigue	73 (39.9)
Anorexia	69 (37.7)
Chest pain	66 (36.1)
Headache	62 (33.9)
Nausea	56 (30.6)
Chills	43 (23.5)
Muscle pain	33 (18.0)
Vomiting	30 (16.4)
Night sweating	29 (15.8)
Abdominal pain (in general)	28 (15.3)
Diarrhoea	26 (14.2)
Loss of weight	25 (13.7)
Confusion	19 (10.4)
Disorientation/attention deficits	16 (8.7)
Haemoptysis	15 (8.2)
Joint pain	8 (4.4)
Neurological signs	8 (4.4)
Eye problems	5 (2.7)
Sore throat	4 (2.2)
Skin rash	2 (1.1)
Jaundice	2 (1.1)
Neck stiffness	2 (1.1)

**Table 5:** Radiologic findings, laboratory tests, treatment, and follow-up of hospitalized acute Q fever patients (n = 183).

	Hospitalized acute Q fever patients (n = 183)	Missing data
	n (%)	n
**Chest x-ray**		4
No infiltrate	18 (10.1)	
Unilateral infiltrate	138 (77.1)	
Bilateral infiltrate	16 (8.9)	
Pleural effusion	4 (2.2)	
Ambiguous	3 (1.7)	
**Laboratory tests at admission**		
Elevated CRP (>10 mg/L)	169 (94.4)	4
Accelerated ESR^a^	163 (89.6)	1
Leukocytosis (>10.0×10^9^/L)	60 (32.8)	
Leukopenia (<4.0×10^9^/L)	1 (0.5)	
Thrombocytosis (>400×10^9^/L)	14 (7.7)	
Thrombocytopenia (<150×10^9^/L)	6 (3.3)	
Anaemia (<8.5 mmol/L)^b^	59 (32.2)	
Elevated bilirubin (>17 µmol/L)	42 (25.6)	19
Highly elevated bilirubin (>34 µmol/L)	2 (1.2)	19
Elevated ALT (>45 U/L)^b^	54 (32.3)	16
Highly elevated ALT (>90 U/L)^b^	15 (9.0)	16
Gamma GT (>55 U/L)^b^	74 (44.0)	15
Hyponatraemia (<135 mmol/L)	89 (50.9)	8
Mild hyponatraemia (130–134 mmol/L)	77 (44.0)	
Moderate hyponatraemia (120–129 mmol/L)	12 (6.9)	
Severe hyponatraemia (<120 mmol/L)	0 (0.0)	
Hypokalaemia (<3.5 mmol/L)	42 (24.1)	9
**Treatment**		
Adequate antibiotic treatment started^c^	155 (84.7)^c^	
Immunosuppressive drugs during admission	21 (11.5)	
**Follow-up** d		
ICU admission	9 (4.9)	
Mortality^e^	11 (6.0)^e^	
Serological profile indicative of chronic Q fever in follow-up (IgG phase I≥1∶1,024) [12]	17 (9.3)	
** **Proven chronic Q fever	4 (2.2)	
** **Probable chronic Q fever	2 (1.1)	
** **Possible chronic Q fever	11 (6.0)	

ALT: alanine aminotransferase; CRP: C-reactive protein; ESR: erythrocyte sedimentation rate; Gamma GT: Gamma glutamyl transferase; ICU: Intensive Care Unit.

a Male <50 years of age: >15 mm/h; male≥50 years of age: >20 mm/h; female <50 years of age: >20 mm/h; female≥50 years of age: >30 mm/h.

b Values presented are applicable to male patients. Female: anaemia (hemoglobin): <7.5 mmol/L; elevated ALT: >35 U/L; highly elevated ALT: >70 U/L; Gamma GT: >40 U/L.

c Defined as doxycycline, 200 mg/day; moxifloxacin, 400 mg/day; ciprofloxacin, 1,000 mg/day per oral dose [22]. Adequate treatment during or after hospitalization (medication used for at least 10 days): 113/155 (72.9%) of patient who started adequate treatment, 15/155 (9.7%) received adequate antibiotics for less than 10 days, in 27/155 (17.4%) duration unknown/not reported in clinical patient files.

d Within two years after hospital admission.

e All-cause mortality within two years after hospitalization. All patients had underlying disease. Two patients died at the intensive care unit during hospital admission. The eleven deceased patients include one proven and one possible chronic Q fever case. The chronic infection might have contributed to the death in the proven chronic Q fever patient, though also other underlying illnesses were present.

Almost all patients had elevated C-reactive protein (CRP) levels (>10 mg/L); median 182 mg/L (range 0–672; IQR 107–247). Upon admission, trombocytopenia and trombocytosis were present in 3.3% and 7.7% of cases, respectively, but no consistent pattern in resolution of these abnormalities was observed. Liver chemistry tests at admission were available for 167/183 (91.3%) patients, and abnormal values (i.e., ALT; males >45 U/L, females>35 U/L) were seen in 32.3% (Table 5). Bilirubin was elevated in 25.6% of the admitted patients. None of the patients met our definition of hepatitis. No liver biopsies were performed. Hyponatraemia (<135 mmol/L) was found in 50.9%; the majority had mild hyponatraemia (77/89; 86.5%), and severe hyponatraemia was not observed. A lumbar puncture was performed in five patients: three were suspected of meningitis (none confirmed), and two of Guillain-Barré syndrome (both confirmed). Ten (5.5%) patients needed to be re-hospitalized within six weeks after they had been initially discharged.

Heart failure (11.5%) and chronic obstructive pulmonary disease (COPD) (n = 19; 10.4%) were the most common co-morbidities in hospitalized acute Q fever cases (Table 2). Pregnancy or infection with HIV/AIDS was not observed. One underlying condition was reported for 38 (20.8%) patients, two or more co-morbidities for 37 (20.2%). Eighty-two patients (47.1%) were smokers, which is a higher percentage than observed in the general Dutch population (26.3%) [27].

Eleven (6.0%) patients, all with underlying illness, died within two years after hospital admission due to different causes, of whom two (1.1%) died in the intensive care unit during their hospital admission (one with disseminated malignancy and one with *Staphylococcus aureus* sepsis) (Table 5). In one deceased patient, the proven chronic Q fever infection might have contributed to the death, the other ten had other causes of death. The median number of weeks between the date of admission and death was 23 weeks (range 1–97) and the median age at death was 76 years (range 63–86). Deceased patients were significantly older than patients still alive two years after hospitalization (*p*<0.001).

One or more sequelae following acute Q fever were reported for 81 of 153 (52.9%) patients with available information. The most common complaints were fatigue (n = 55; 67.9% of patients with sequelae), dyspnoea (n = 11; 13.6%), and decreased physical fitness (n = 7; 8.6%). Other complaints recorded for no more than four patients were: fever, chronic cough, neurological sequelae, headaches, thoracic pain, depression, and sweating. Seventeen patients (9.2%) developed chronic Q fever during follow-up (four proven, two probable, and eleven possible chronic Q fever cases - Table 4).

### Pneumonia patients

In a study on CAP patients conducted by van Gageldonk-Lafeber *et al.* [23], 339 CAP patients were included. We excluded 48 patients because of they had *C. burnetii* pneumonia and another 37 patients who were not hospitalized. The features of the remaining 254 hospitalized CAP patients, the bacterial aetiology pneumonia subgroup (n = 104), and the 154 hospitalized Q fever patients with an infiltrate on their chest x-ray are listed in Table 6. Hospitalized Q fever-related pneumonia patients were significantly younger than hospitalized CAP patients (median age at admission 56 vs. 68 years, respectively, *p*<0.001). The median PSI score was significantly lower (*p*<0.001) in hospitalized acute Q fever patients (median 58, range 0–142) than for the overall group of CAP patients (median 88, range 21–184) and the subgroup of hospitalized CAP patients diagnosed with another bacterial infection (median 86, range 27–184). The odds of being hospitalized with mild pneumonia (PSI I–III) was significantly higher for Q fever patients than for other CAP patients (OR 3.69, 95% CI 2.26–6.04), and also compared to CAP patients diagnosed with another bacterial infection (OR 3.22, 95% CI 1.79–5.81). When using the CURB-65 score, similar results are found: mild pneumonia (CURB-65 0–1) in the hospitalized Q fever group compared with CAP patients (OR 4.99, 95% CI 3.14–7.94) and CAP patients diagnosed with another bacterial infection (OR 5.49, 95% CI 3.12–9.67).

**Table 6:** Comparison of hospitalized acute Q fever pneumonia patients (n = 154) with patients admitted with a community-acquired pneumonia (CAP) (n = 254) and with CAP patients with bacterial aetiology other than *C. burnetii* (n = 104).

	Q fever pneumonia (n = 154)	Community-acquired pneumonia (n = 254)	*p*-value	Bacterial pneumonia (n = 104)	*p*-value
	n (%)	n (%)		n (%)	
Male	98 (63.6)	155 (61.0)	0.598^a^	68 (65.4)	0.774^a^
Median age [IQR] (years)	56 [42–65]	68 [54–76]	<0.001^b^	67 [55–76]	<0.001^b^
<40 years	34 (22.1)	25 (9.8)		11 (10.6)	
40–59 years	61 (39.6)	59 (23.2)		21 (20.2)	
60–79 years	49 (31.8)	127 (50.0)		55 (52.9)	
≥80 years	10 (6.5)	43 (16.9)		17 (16.3)	
Smoker^c^	71 (48.3)	62 (31.3)	0.001^a^	31 (38.8)	0.167^a^
Median duration of admission [IQR] (days)^d^	5 3–7	8 5–13	<0.001^b^	8 6–14	<0.001^b^
Median duration between onset of illness and admission [IQR] (days)^e^	4 3–7	4 1–7	0.325^b^	4 2–7	0.522^b^
**Medical history**					
COPD^f^	16 (10.4)	83 (38.1)	<0.001^a^	41 (47.7)	<0.001^a^
Malignancy^g^	13 (8.4)	21 (9.4)	0.745^a^	12 (13.2)	0.236^a^
**PSI score** h **^,^** i			<0.001^j^		<0.001^j^
I	56 (36.4)	33 (14.7)		8 (8.8)	
II	45 (29.2)	43 (19.1)		22 (24.2)	
III	26 (16.9)	50 (22.2)		24 (26.4)	
IV	26 (16.9)	76 (33.8)		26 (28.6)	
V	1 (0.6)	23 (10.2)		11 (12.1)	
Median score [IQR]^i^	58 [41–83]	88 [65–111]	<0.001^b^	86 [68–116]	<0.001^b^
**CURB-65 score** k **^,^** l			<0.001^j^		<0.001^j^
0	86 (55.8)	48 (21.1)		17 (18.5)	
1	34 (22.1)	46 (20.3)		19 (20.7)	
2	26 (16.9)	79 (34.8)		34 (37.0)	
3	5 (3.2)	44 (19.4)		20 (21.7)	
4	3 (1.9)	9 (4.0)		2 (2.2)	
5	0 (0.0)	1 (0.4)		0 (0.0)	
Severe pneumonia^l^^,^ m	45 (29.2)	145 (63.9)	<0.001^a^	61 (66.3)	<0.001^a^

IQR: interquartile range; COPD: chronic obstructive pulmonary disease.

a Chi-square test.

b Mann-Whitney U test.

c Information missing or unknown for seven Q fever pneumonia patients, 56 CAP patients and 24 bacterial pneumonia patients.

d Three CAP patients who died on the same day as visiting the emergency department not included, two patients not included in the bacterial pneumonia group.

e Information missing for three Q fever pneumonia patients, 46 CAP patients and 19 bacterial pneumonia patients.

f Information missing or unknown for 36 CAP patients and 18 bacterial pneumonia patients.

g Information missing or unknown for 31 CAP patients and 13 bacterial pneumonia patients.

h Pneumonia Severity Index (PSI): risk class: I–III = low; IV = moderate; V = severe.

i Information missing for 29 CAP patients and 13 bacterial pneumonia patients.

j Chi-square test for trend.

k Confusion, blood Urea nitrogen, Respiratory rate, Blood pressure, age≥65 (CURB-65) score: 0–1 = mild pneumonia; 2 = moderate pneumonia; 3–5 = severe pneumonia.

l Information missing for 27 CAP patients and 12 bacterial pneumonia patients.

m Severe pneumonia is defined as PSI risk class ≥IV and/or CURB-score ≥2.

### Environmental exposure

The median distance from the home address of hospitalized acute Q fever patients to the closest infected small ruminant farm was 4,330 m (IQR 1,990–5,375 m), while non-hospitalized acute Q fever patients (n = 1,545) lived at a median distance of 3,555 m (IQR 1,841–5,237 m; *p* = 0.169). Logistic regression showed no significant increase in risk for hospitalization for people living closer (0–2,000 m or >2,000–5,000 m) to an infected goat or dairy sheep farm than people living further away (i.e., ≥5,000 m).

## Discussion

Remarkable differences in clinical presentation were observed between hospitalized *C. burnetii* pneumonia patients and patients hospitalized for pneumonia with another aetiology. In general, hospitalized acute Q fever patients were younger with lower pneumonia severity scores and less co-morbidity, and the duration of their admission was shorter. This might be explained by the severe subjective symptoms of pneumonia, mainly dyspnoea, witnessed by treating physicians in hospitalized Q fever patients (personal communications). As subjective dyspnoea is not included in the pneumonia severity scoring systems, this may be a reason for hospital admission, despite the low severity score. Also the discrepancy between low pneumonia severity scores and abnormalities on the chest x-ray has been associated with hospitalization [28]. While in the Netherlands, the large majority of CAP patients (97%) are treated by their GPs as outpatients [29], all but one of the hospitalized acute Q fever patients were admitted through the hospital emergency department. This suggests that self-referral or referral to the emergency department by the GP of otherwise relatively healthy patients might also be related to the high hospitalization rate.

In 2007, the epidemic was confined mainly to Bernhoven Hospital's catchment area and we presume the high admission rate to be explained in part by unfamiliarity with acute Q fever at the beginning of the epidemic, and retrospective active case finding among hospitalized pneumonia patients. In 2008 and 2009, when the epidemic was spreading, admission rates in Bernhoven Hospital were lower than reported nationwide for notified cases [4], although still higher than the 2–5% found in France [2], [3]. However, from other reported outbreaks elsewhere, large variations in hospitalization rates have been reported, for example 8% and 25% in Germany [16], [30], 37% in Serbia [31], and 60% in the UK [32]. It has been suggested that a high dose of *C. burnetii* bacteria or a prolonged continuous exposure to it might lead to a more severe clinical presentation [15], [16], [17], which was not confirmed by our analysis of proximity to an infected farm. Higher awareness and familiarity with acute Q fever in Bernhoven Hospital and adequate public health information may play a role in Dutch regional hospitalization rate differences, which is currently under investigation. In addition, later in the epidemic diagnostic tests for Q fever were done more frequently and more rapidly requested [4], [5], while diagnostic facilities improved through the introduction of PCR [13].

We found moderately elevated liver chemistry tests (ALT or bilirubin) in hospitalized acute Q fever patients, but no cases of hepatitis, although liver biopsies were not performed. Nevertheless, elevated liver enzymes are a common feature of acute Q fever infections [2], [8], [33]. The case definition of hepatitis differs between studies [1]. In addition, the majority of the patients with elevated liver chemistry tests had pneumonia. In contrast to our findings, hepatitis seems to be the major clinical presentation in France [2], [7], [8] and southern Spain [9]; while in the Spanish Basque region pneumonia predominates [6]. The reasons for this geographical variation may include differences in the route of the infection (aerosol or ingestion) [8], [34], host factors [3], differences in *C. burnetii* strains [2], and the infectious dose level [15], [17].

Furthermore, hyponatraemia was more common in our study population (50.9%) than described previously for CAP and acute Q fever patients (27.9% and 28.2%, respectively) [10], [35]. Hyponatraemia is the most common electrolyte imbalance observed in hospitalized patients, and has been associated with prolonged hospital stay [36], [37] and increased in hospital mortality in hospitalizations for all causes [37], and for pneumonia hospitalizations [35]. However, other studies reviewing pneumonia patients reported no difference in hospital mortality [36], [38].

While the clinical presentation of hospitalized acute Q fever patients was severe enough to be hospitalized, the median hospital stay of just five days suggests that the duration of symptoms was generally short. Typically, they were relatively young, male, had little co-morbidity, and were smokers, which is in accordance with previous studies [3], [8], [10], [39], [40]. Low CURB-65 scores in acute Q fever patients have also been reported before [28]. Reported clinical symptoms were mostly in line with the literature [2], [10], [39]. Relative bradycardia was also observed, which has been described previously [2], [21], [41], [42]. We observed two cases of Guillain-Barré syndrome – which is a rare, but previously described complication of acute Q fever [7], [43], [44]. Additional microbiological testing revealed the presence of antibodies against *M. pneumoniae* in 22 patients, although only two met our definition, which required an antibody titre≥1∶320. Overlapping positive *M. pneumoniae* and *C. burnetii* serologic results have been described before [45]. Adequate antibiotic treatment was started during or after hospitalization in 84.7% of patients, which is higher than what was found nationwide among patients treated by their GP or hospital physician in 2007 or 2008, when 60.3% and 72.2% received adequate treatment, respectively [22]. Presumably, this reflects higher awareness of, and familiarity with, acute Q fever in Bernhoven Hospital. An acute Q fever-related mortality rate of 1.1% was found, which is in accordance with previous findings [46], [47].

The diagnosis of chronic Q fever relies on high IgG phase I titres in combination with a clinical evaluation and detection of *C. burnetii* DNA [12]. Seventeen (9.2%) patients developed a serological profile indicative of chronic Q fever, though the majority only had high IgG phase I titres (≥1∶1,024) and no risk factors for a chronic Q fever infection, indicating a possible chronic Q fever infection according to the Dutch Q fever Consensus Group [12]. Only four (2.2%) patients had a proven chronic Q fever infection which is similar to overall conversion rates reported in literature (0–5%), though comparison with data from literature is hampered by the use of different case definitions [48].

Though exposure dose is established as an individual risk factor for infection with *C. burnetii* [14], distance to the nearest positive Q fever ruminant farm as a measure of exposure dose was not associated with hospitalization. This corresponds with studies that found a high prevalence of antibodies against *C. burnetii* among the occupationally exposed, such as goat and sheep farmers and veterinarians [4], [49], with relatively low levels of clinical illness suggesting that the antibodies are protective against symptomatic Q fever [4].

The strength of this study is that we were able to describe a large cohort of consecutively admitted patients in one hospital in one specific epidemic. Most of the collected data were complete and follow-up data were available for the majority of patients.

A limitation of this study is the absence of a pre-defined control group. In the database of the non-hospitalized *C. burnetii* infected patients, information on most clinical characteristics were absent, as the patients were treated by their GP. In order to compare the clinical characteristics, we used CAP patients from a historical study group [23]. Yet, all these patients were diagnosed within the same region with the same protocols for diagnostic procedures and treatment, and within the same time period. Secondly, not all items of the pneumonia severity scores were available in the medical records. This means that the observed scores sometimes actually might have been higher. However, the low CURB-65 scores we found in acute Q fever patients have been reported before [28].

In conclusion, hospitalized Dutch acute Q fever patients mostly presented with fever and pneumonia, while hepatitis was not observed. Patients with acute Q fever pneumonia were hospitalized despite young age, low PSI and CURB-65 scores, and limited co-morbidity, presumably because of severe subjective symptoms in disease presentation. Proximity to an infected ruminant farm, reflecting level of exposure to *C. burnetii*, does not influence admission rates.
